# An evolutionary consequence of dosage compensation on *Drosophila melanogaster *female X-chromatin structure?

**DOI:** 10.1186/1471-2164-11-6

**Published:** 2010-01-05

**Authors:** Yu Zhang, Brian Oliver

**Affiliations:** 1 Laboratory of Cellular and Developmental Biology, National Institute of Diabetes and Digestive and Kidney Diseases, National Institutes of Health, Bethesda MD 20892-8028 USA

## Abstract

**Background:**

X chromosomes are subject to dosage compensation in *Drosophila *males. Dosage compensation requires *cis *sequence features of the X chromosome that are present in both sexes by definition and *trans *acting factors that target chromatin modifying machinery to the X specifically in males. The evolution of this system could result in neutral X chromatin changes that will be apparent in females.

**Results:**

We find that the general chromatin structure of female X chromosomes is distinct from autosomes. Additionally, specific histone marks associated with dosage compensation and active chromatin marks on the male X chromosome are also enriched on the X chromosomes of females, albeit to a lesser degree.

**Conclusions:**

Our data indicate that X chromatin structure is fundamentally different from autosome structure in both sexes. We suggest that the differences between the X chromosomes and autosomes in females are a consequence of mechanisms that have evolved to ensure sufficient X chromosome expression in the soma of males.

## Background

*Drosophila *X chromosomes show peculiar features in both gene expression [1] and gene evolution [2]. One of the most striking consequences of X chromosome hemizygosity in males, is dosage compensation, a process which brings X chromosome and autosome expression into balance [3-5]. Dosage compensation was probably acquired gradually in the course of sex chromosome evolution, as sex chromosomes are thought to arise by divergence of an ancestral autosome pair [6]. Gene loss from the Y chromosome creates an increasingly aneuploid condition in males and is thought to be the driving force in the evolution of global X-chromosome dosage compensation. In the absence of dosage compensation genomic imbalance results in male lethality.

It has long been known that selective pressures applied to just one of the sexes can effect change in the other [7]. For example, the coloration of certain birds or the nipples of mammals are advantageous to one of the sexes and are likely to be present as an evolutionary side-effect in the other. X chromosome dosage compensation might also show evidence of this type of sexual selection. X chromosome dosage compensation requires both *cis *and *trans *components [1]. *Cis *changes resulting from selection of the compensation system in males will also be present in females, and might alter the character of the X chromosome in females as a secondary consequence [8]. Indeed, we have previously noted a slight over-expression of both male and female X chromosomes relative to autosomes [4,9], which suggests that the X chromosome is inherently more active than autosomes. We have therefore examined the structure of X chromatin in females in detail.

Expression patterns and especially X chromosome dosage compensation are mediated by chromatin modification [1,10,11]. Histones are nucleosome subunits required for packing DNA into the confines of the nucleus. It has long been know that chromatin structure changes are associated with transcription [12]. For example, when chromatin is physically sheared to small fragments by sonication or enzyme digestion, shearing-bias is associated with different chromatin structures across the genome [13-15]. Histones are also modified on N-terminal tail residues to generate an expanding repertoire of histone modifications that are important modulators of transcription [16,17]. It has become increasingly clear that specific types of modification are associated with particular transcriptional outcomes. For example, acetylation events are broadly associated with transcriptional activation, while methylation events can have either activating or repressing roles.

One of the best studied histone modifications is the acetylation of Histone 4 on Lysine 16 (H4K16ac). In organisms from yeast to humans, H4K16ac is broadly associated with active genes, and the Histone Acetyl Transferase (HAT) that writes the modification is required for viability [18-21]. In *Drosophila*, H4K16ac is highly enriched on the X chromosomes of males [22,23], and the responsible HAT, Males Absent on First (Mof), is required for male viability [21]. While Mof is associated with some genes in both males and females [24], Mof is greatly enriched on the male X chromosome due to targeting by the male-specific-lethal (MSL) complex. MSL is composed of proteins (Mle, Msl1, Msl2, Msl3, and Mof) and two non-coding RNAs encoded on the X (RoX1 and RoX2) [1]. It is thought that the greatly increased H4K16ac levels act to increase X chromosome expression in males, although it is also possible that X chromosome enrichment depletes autosomes of H4K16ac [1,25]. In either model, X chromosome and autosome expression are equilibrated to restore transcription balance.

Another chromatin modifying enzyme, Jil1, is also enriched on the X chromosome of males [26-28]. This kinase mediates phosphorylation of Histone 3 at serine 10 (H3S10ph). Jil1 is required for full dosage compensation and associates with the MSL complex [27,29]. H3S10ph is implicated in both chromosome condensation during mitosis and transcriptional activation during interphase, suggesting that Jil1 has more general roles in addition to dosage compensation. Another mark associated with active transcription, dimethylation of histone H3 at lysine 4 (H3K4me2) [17] is general, and thus likely to be MSL complex independent.

We have performed chromatin-shearing experiments showing that X chromatin differs from autosomal chromatin in both males and females. Additionally, the histone marks associated with X chromosome dosage compensation in males are modestly enriched on female X chromosomes. These data indicate that X chromatin is distinct even in the absence of dosage compensation. We suggest that the pattern in females is a tolerated neutral side-effect of the evolution of X chromosome dosage compensation in males.

## Results

### Global analysis of chromatin structure in females and males

We took advantage of differential shearing to probe chromatin structure by deep DNA sequencing (DNA-Seq) [30]. Specifically, we sheared cross-linked chromatin, size selected for short (200 bp) fragments, performed deep sequencing, and aligned the DNA reads to the reference genome. Because these sequencing reactions generated reads from the ends of size-selected fragments, increased mapped read density occurs in regions of preferential shearing.

**Figure 1:**
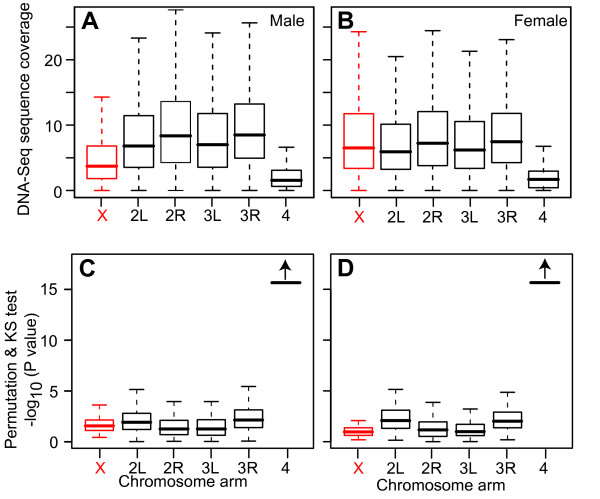
**Sheared chromatin DNA-Seq coverage in male and female adult flies**. (A-B) Box plots of the distributions of average DNA-Seq sequence coverage (in non-overlapping 1 kb windows) in male (A) and female (B) adult flies separated by chromosome arms (X chromosome in red). 25th to 75th percentiles (boxes), medians (lines in boxes), and ranges (whiskers, 1.5 times the interquartile range extended from both end of the box ) are indicated for each chromosome. Significance of the chromosome arm distributions relative to the whole genome data set as determined by permutation sampling and KS test (C, D).

We constructed libraries of DNA from sheared chromatin from female and male adult flies and obtained about 3 million uniquely mapping 35 bp reads for each sex. We then parsed the mapped reads by chromosome arms. The X chromosome and each arm of the 2^nd ^and 3^rd ^chromosomes bear about 20% of the genome, while the 4^th ^chromosome bears <1%. The average coverage of the different chromosomes (depth of sequence coverage at each base in non-overlapping 1 kb windows normalized by total sequenced base pairs) was similar (average coverage from 6.81-8.49 for chromosome arms in males and 5.91-7.23 in females) except the X chromosome in males (average coverage 3.71) and the 4^th ^chromosome in both males and females (average coverage 1.54 and 1.68) (Figure 1A, B). The average coverage of the male X chromosome was 48% of the autosome coverage, which is consistent with the male karyotype. Permutation testing of chromosome arm sequence coverage relative to genome-wide coverage showed that the 4^th ^chromosome had unusually low read density (Figure 1C, D). The under-representation of DNA sequence coverage on the heterochromatin-rich, gene poor, the 4^th ^chromosome [31] suggests that this chromosome was resistant to shearing. Thus, the 4^th ^chromosome data suggests that lower sequence coverage is associated with a more "closed" chromatin state in this largely heterochromatin chromosome.

**Figure 2:**
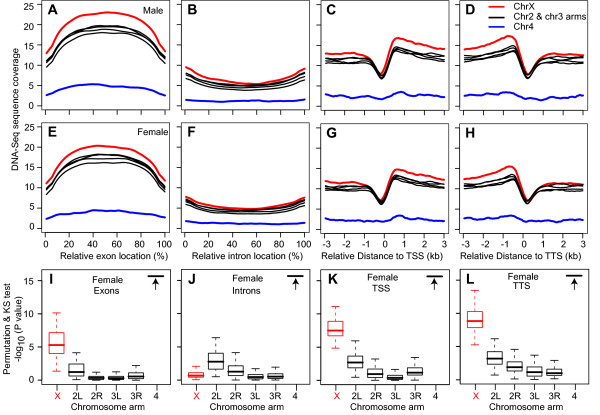
**DNA-Seq coverage profiles of X chromosome and autosome gene features**. (A-H) The average sequence coverage profiles of exon/intron regions and regions near transcription start sites (TSS) and termination sites (TTS) of all annotated gene models on the X chromosome (red), major autosomes (black), and the dot chromosome 4 (blue) in male (A-D) and female (E-H) adult flies. Male X chromosome coverage was multiplied by two to correct for karyotype. Significance of the chromosome arm distributions relative to the whole genome data set as determined by permutation sampling and KS test for females (I-L). P Values < 10^-15 ^are off-scale (black arrow).

We were interested in shearing at gene models, as chromatin alternations associated with transcription, and thus dosage compensation, should be evident at those sites. To summarize the gene model read density patterns genome-wide, we calculated read coverage at multiple gene model features. We clearly observed higher sequence coverage at exonic regions relative to intronic regions and elevated coverage flanking transcription start and termination sites on X chromosomes and autosomes (Figure 2A-H). We suggest that these signatures are due to association of different protein-complex types during transcription. Thus, like DNase hypersensitivity [32], mechanically sheared chromatin breaks preferentially at some sites to provide information about chromatin structure genome-wide.

We then compared the coverage profiles of X chromosome and autosome gene model features *inter se *to determine if the structure of the X chromosome differed from the autosomes. Indeed, the average coverage for X chromosome genes was clearly higher than autosome arm genes at exons, transcription start sites, and transcription termination sites in both males and females (Figure 2A-H). The 4^th ^chromosome always showed greatly reduced sequence coverage. Permutation testing clearly suggests that the X chromosome shearing at exons, transcription start sites, and termination sites is significantly greater than the pattern genome-wide in both males (not shown) and females (Figure 2I-L). These data indicate that X chromosome genes are susceptible to shearing, probably as result of a more open structure.

### Distribution of histone marks on female and male chromosomes

**Figure 3:**
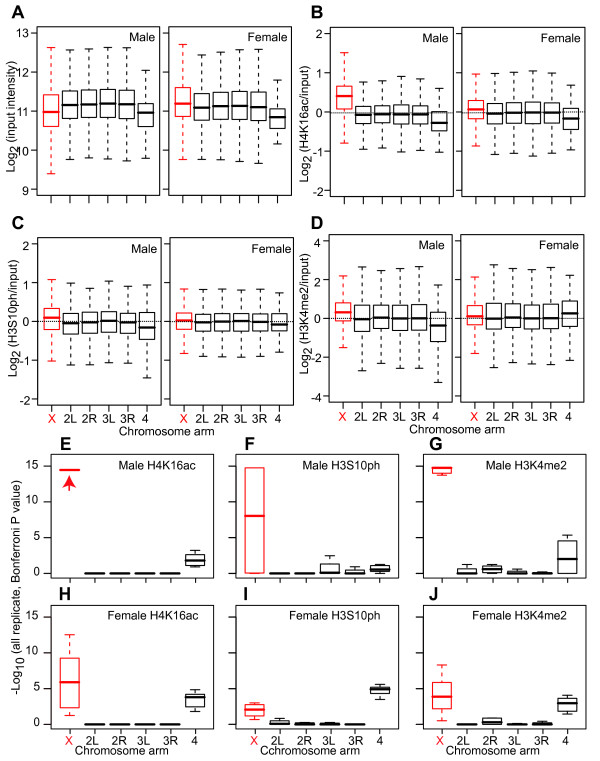
**Histone mark distributions**. Box plots of ChIP input DNA intensities (log_2_) (A), H4K16ac ChIP/input ratios (log_2_) (B), H3S10ph ChIP/input ratios (C) and H3K4me2 ChIP/input ratios (D) in male (left) and female (right) adult flies separated by chromosome arms (X chromosome in red). See Figure 1 for box plot format. (E-J) The distributions of ChIP/input ratios for chromosome arms were compared inter se. Box plots of Bonferroni corrected P values from KS tests of ChIP/input ratios in all ChIP-chip replicates in male (E-G) and female (H-J) adult flies are shown. See Figure 1 for box plot format. P Values < 10^-15 ^are off-scale (red arrow).

If *cis *features have evolved on the X chromosome to facilitate dosage compensation in conjunction with the *trans*-acting MSL complex, then the X chromosome in females might show a "shadow" profile resembling the male X chromosome. We therefore more specifically probed for the histone modifications that are associated with X chromosome dosage compensation. To localize histone marks in the genome, we carried out chromatin immunoprecipitation coupled with microarray hybridization (ChIP-chip) to FlyGEM arrays on sex-sorted adult flies. The DNA from ChIP and input chromatin were hybridized to the same slides, normalized, and averaged to generate a ratio for each gene (see methods). In agreement with the DNA-Seq coverage data, the intensity of ChIP input X chromosome DNA was significantly higher than the input for the autosomes in females and was significantly lower for the 4^th ^chromosome (Bonferroni corrected KS test, Figure 3A). Thus, the input data also suggest that X chromatin is more open. ChIP ratios (ChIP enriched DNA/input) eliminate the effect of chromatin on the input channel when testing for histone modification enrichment.

As expected, we found a strong enrichment for H4K16ac on the male X chromosome relative to male autosomes (Figure 3B, E). The average H4K16ac ChIP ratio (ChIP enriched DNA/input) on male X chromosome genes was 1.39 fold higher than the value of all autosome gene. These data show that we are able to easily score the high levels of H4K16ac present on the male X chromosomes in adult flies. Interestingly, we observed a modest (1.06 fold) but significant enrichment for H4K16ac on female X chromosomes relative to all the autosomes (Figure 3B, H). In both males and females, the ratio of H4K16ac ChIP/input was similar for all major autosome arms (2L, 2R, 3L, 3R) indicating that there are no differences in H4K16ac among the major autosome arms within either sex. These data indicate that not only is X chromatin different from autosomal chromatin in females, but that the important dosage compensation mark H4K16ac is generally enriched on X chromosomes.

We observed a modest, but significant 1.09 fold enrichment for the H3S10ph mark on the male X chromosome relative to the autosomes (Figure 3C, F). We also observed a 1.02 fold enrichment of H3S10ph on the female X chromosome, but this difference was only modestly significant (Figure 3C, I). As observed for the other two histone marks, we found a significant enrichment for H3K4me2 on the X chromosome in males (Figure 3D, G). But again, we also found significant enrichment for H3K4me2 on the female X chromosome (Figure 3D, J). Thus, the patterns of histone mark accumulation on the male X chromosome appear to be shadowed by similar distributions on the female X chromosome.

### Distributions of histone marks correlate with each other and active genes

**Figure 4:**
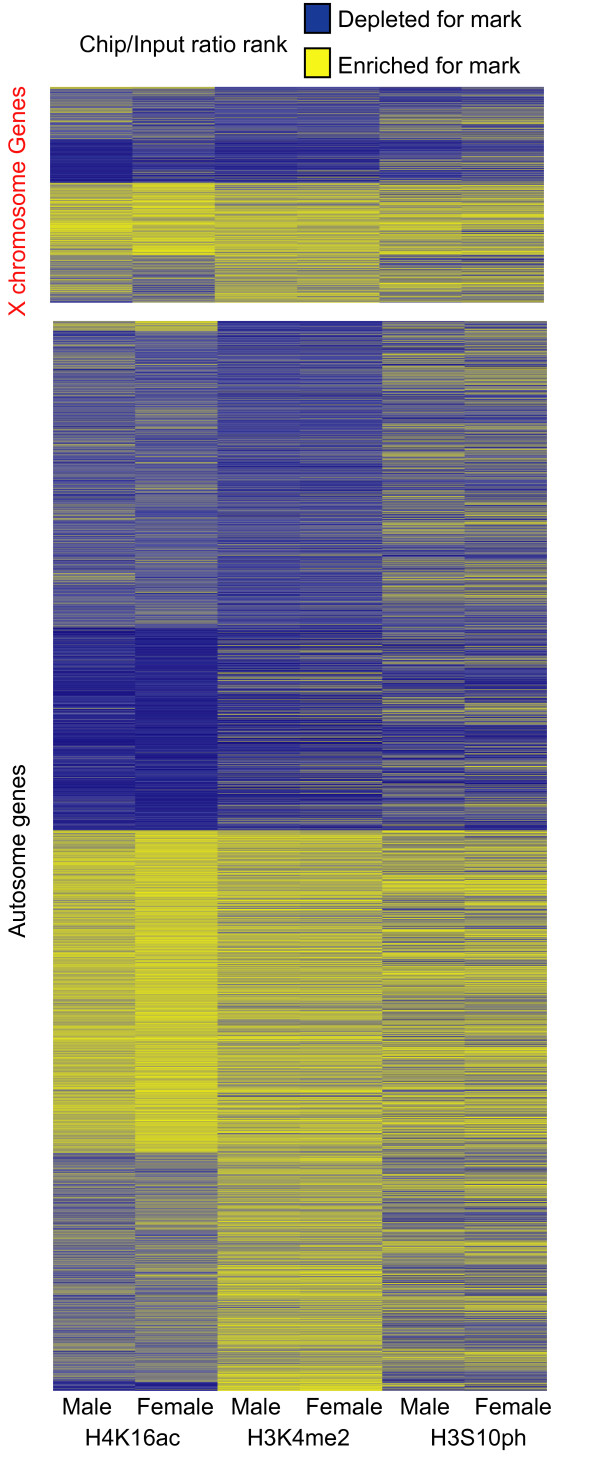
**Correlation among histone marks**. K-means clustering (k = 2) of rank orders for ChIP enriched DNA/input ratios of individual histone marks in male and female adult flies. X chromosome genes (top) and autosome genes (bottom) are clustered separately. The order of ChIP samples was fixed as indicated in the figure. Genes enriched for histone marks (yellow) and genes depleted for histone marks (blue) are indicated.

If the female X chromosome shadows the pattern observed on the male X because of underlying changes in X chromosome sequence, then the histone marks should also correlate at the gene level. To determine if the same genes show similar histone modifications on both the male and female X chromosome, we directly examined correlations by using the rank order of individual histone marks to normalize the results of the three distinct ChIP data sets and then subjected the rank order data to cluster analysis (Figure 4). To highlight associations on the autosomes, we clustered the X chromosomes and autosomes separately. We observed a strong positive correlation between H4K16ac and the other two histone marks on both X chromosomes and autosomes. For example, Spearman's Rank Correlation indicates that H4K16ac on male X chromosomes was positively correlated with H3K4me2 (Spearman's Rho = 0.64) and H3S10ph (Spearman's Rho = 0.4). On the autosomes, H4K16ac enrichment was positively correlated with female H4K16ac (Spearman's Rho = 0.69) and with other two histone modifications (Spearman's Rho ranged from 0.40-0.49). More importantly, there was a good rank correlation between male and female X chromosomes for H4K16ac (Spearman's Rho = 0.46), H3S10ph (Spearman's Rho = 0.68), and H3K4me2 (Spearman's Rho = 0.92). These data indicate that X chromosomes in the sexes differ by degree of modification, but the pattern of modifications along the male X chromosome is shadowed along the female X chromosome.

**Figure 5:**
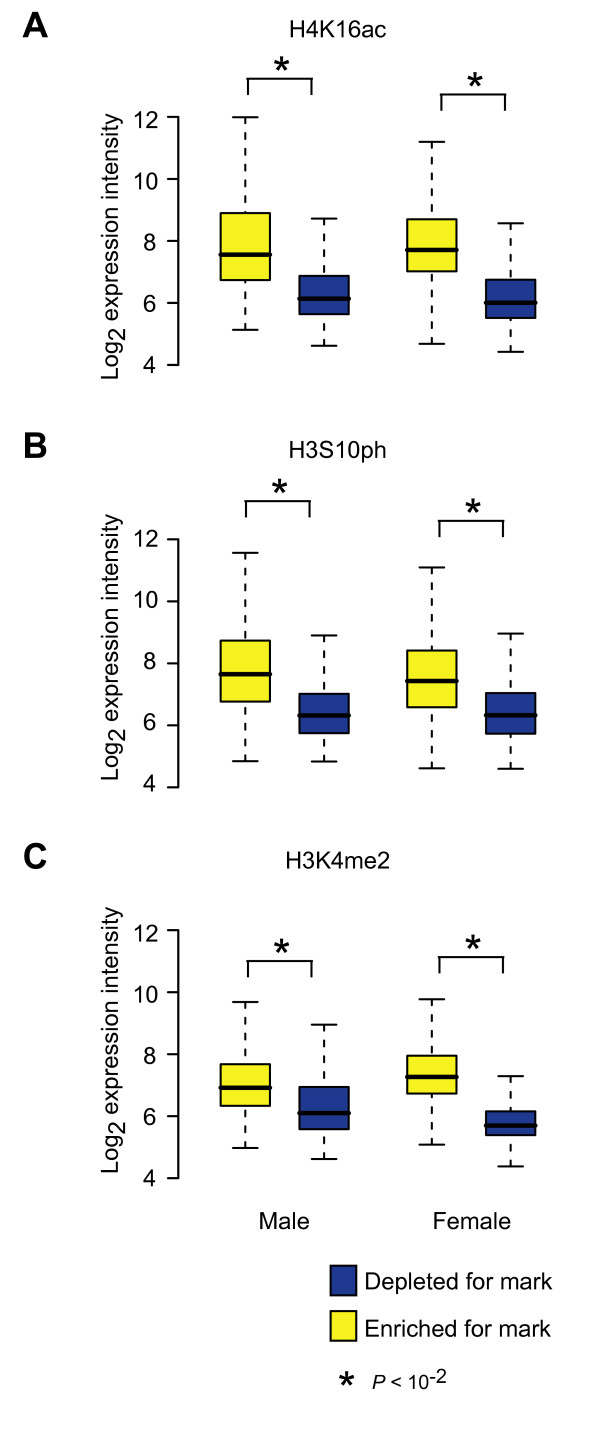
**Correlation between marks and expression**. Box plots of expression intensities (log_2_) of male or female adult flies for genes enriched for H4K16ac (A), H3S10ph (B), H3K4me2 (C) (ChIP/input percentile rank >90%) and genes depleted for those three histone marks (ChIP/input percentile rank <10%) in male or female adult flies. See Figure 1 for box plot format. Significance differences (P < 10^-2^, Bonferroni corrected KS test) between genes enriched and depleted for histone marks are indicated (asterisks).

Dosage compensation alters transcription relative to autosomes. Therefore, one might expect that genes showing particular modifications in males and females would be expressed. To determine if the three histone marks were associated with transcribed genes, we examined gene expression data for male or female adult flies from a previous study [4] for genes highly enriched or depleted for different histone marks (Figure 5). The genes enriched for any of the three different histone marks all showed significantly higher expression levels compared to genes depleted for histone marks. This observation held in both males and females tissues. For example, the expression level of genes enriched for H4K16ac is 2.68-fold higher in males and 3.77-fold higher in females relative to genes depleted for H4K16ac (*P *< 10^-11^, Bonferroni corrected KS test) within each sex. These data clearly indicate that the three different histone modifications are correlated *inter se *and with expression. We suggest that all three marks contribute to the mild hypertranscription of female X chromosomes.

**Figure 6:**
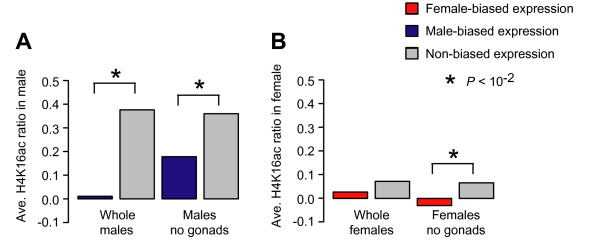
**H4K16ac enrichment and sex-biased expression**. (A) Average H4K16ac ChIP/input ratios (log_2_) in male flies for genes showing male-biased expression (blue) and non-biased expression (grey) in whole flies and in the soma. (B) Average H4K16ac ChIP/input ratio (log_2_) in female flies for genes showing female-biased expression (red) and non-biased expression (grey) in whole flies and in the soma. Significance differences (P < 10^-2^, Bonferroni corrected KS test) between genes with sex-biased expression and genes with non-biased expression are indicated (asterisks).

Germline X chromosomes are dosage compensated by increased expression of the single X in males relative to autosomes [4], but germline dosage compensation is not mediated by the MSL complex [33]. Therefore the histone mark and expression correlations may not hold for the germline. To determine if the shadow marks on the female X chromosome are restricted to genes expressed in the soma, we examined male-biased expression in adult flies, which is dominated by expression within the male germline [2]. If H4K16ac is associated with active transcription only in somatic cells as predicted by our understanding of somatic MSL function, then genes with male-biased expression in whole flies should show reduced H4K16ac levels. Indeed, this is what we observed (Figure 6). X chromosome genes showing male-biased expression in whole flies show very low (average ChIP/input ratio = -0.015) H4K16ac levels in males, compared to X chromosome genes showing non-biased expression in males (average ChIP/input ratio = 0.368, *P *< 10^-12^, Bonferroni corrected KS test). These data support the idea that dosage compensation marks occur at genes active in the male soma, but not the germline.

Dosage compensation may be not being required for genes that should be differentially regulated in the female and male soma. In support of this idea, we also observed lower H4K16ac among X chromosome genes showing male-biased expression in the soma (average ChIP/input ratio = 0.178 for genes with male-biased expression vs. 0.351 for genes with non-biased expression, *P *< 10^-2^, Bonferroni corrected KS test). These data are also consistent with the idea that genes with male-biased expression are poorly expressed on the X chromosome due to limited dosage compensation [34]. Interestingly, the same pattern is observed for genes with female-biased expression. We observed lower H4K16ac among genes showing female-biased expression in females (average ChIP/input ratio = -0.053 for genes with female-biased expression vs. 0.060 for genes with non-biased expression, *P *< 10^-2^, Bonferroni corrected KS test). These data suggest that sex differentially regulated genes are not H4K16ac regulated.

## Discussion

Our data indicate that the dosage compensation marks associated with X chromosomes in males are also enriched on X chromosomes more generally. This is part of a growing body of data showing that the X chromosome is distinct from the autosomes. For example, previous studies show that Jil1 mutants cause accumulation of the H4K9me2 mark and the transcriptional negative regulator HP1 on the X chromosome of both males and females [35]. Additionally, 9 of 29 chromatin associated proteins surveyed in female *Drosophila *Kc167 cells are enriched on the X chromosome [36]. Finally, both Mof and H4K16ac enrichment has been observed on the X chromosomes of female Kc167 cells [24,37], although female results served as controls in those manuscripts and the enrichment in females was not highlighted. Collectively, these data indicate that X chromatin differences from autosomes are not restricted to male-specific dosage compensation. We have previously observed a modest elevation of gene expression from the female X chromosome relative to autosomes in *D. melanogaster *[4] and this same modest (1.11 to 1.24 fold) but highly significant elevation of female X chromosome expression is consistently observed in six other *Drosophila *species [9], suggesting that this modest elevation of expression on X chromosome is independent of microarray platform and species. We suggest that specialized X chromatin contributes to elevated X chromosome gene expression in females in addition to males, albeit to a much more limited extent.

**Figure 7:**
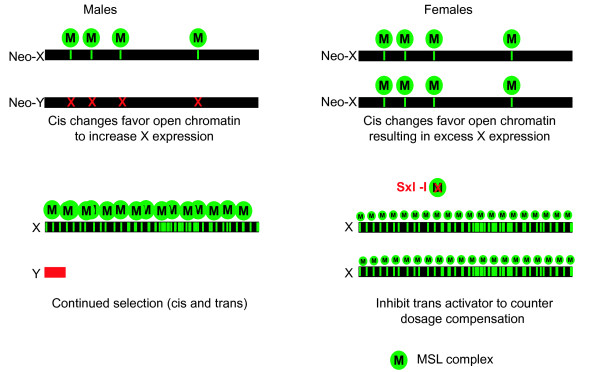
**Model of sex chromosome chromatin structure evolution**.

That there is enrichment for X chromosome marks associated with X chromosome dosage compensation in males is not *de facto *evidence of function within females. Indeed, the viability of female mutants for many of the MSL components, indicates that the MSL complex is not required. It is possible that rather than being functional in females, the observed X chromosome enrichment is a consequence of the evolution of dosage compensation in males [6,38,39]. In the sequence of sex chromosome evolution from an autosome pair, genes are gradually lost from the neo-Y resulting in hemizygosity for those genes in males (Figure 7). Any sequence changes that would promote increased X chromosome expression in males, by for example opening chromatin structure, would be highly advantageous in males. Those sequence changes would also be present in females where they would be disadvantageous. However, if the increase in male fitness does not result in a large negative affect on female fitness, then sequence changes supporting increased epigenetic enhancement of X chromosome expression would be favored. As more genes were lost and the Y chromosome was reduced to a gene desert, the need for balancing expression with the autosomes became critical, such that the general MSL complex became specialized for the X. MSL is highly deleterious for females, but MSL formation is blocked by the action of Sxl on the MSL component Msl2 [1]. We suggest that Sxl activity is sufficient to ensure female viability, but the cis changes on the X chromosome and any low level Mof activity in the absence of MSL result in a more open chromatin structure and slight over-expression from the female X chromosome.

While modest over expression of X chromosome genes in females may be of little evolutionary consequence, one can envision that more extensive over expression would be detrimental. For example, large duplications are poorly tolerated in *Drosophila *and [40] trisomy is poorly tolerated in humans [41]. If the X chromosome tends to be in a favourable state for transcription in both males and females, this could ultimately lead to counter measures in females to prevent functional tetrasomy. This might be what has occurred in mammals, where one X chromosome is inactivated in females. There is recent evidence that the active X chromosomes of mammals show increased expression [4,42,43] raising the possibility that X inactivation is a consequence of X chromosome dosage compensation in males. The main dosage compensation counter measure in *Drosophila *females is inhibition of MSL complex formation by Sxl protein [44]. However, if the observed over expression of X chromosomes in *Drosophila *females [9] was more extreme, then inactivating an X chromosome in females to counter X chromosome dosage compensation in males would be logical. This may be how such a mechanism evolved in mammals.

## Conclusions

In this study we examined the general chromatin structure and multiple histone marks in both *Drosophila *males and females. We found that X chromatin structure is different from autosome structure independent of sex. Histone marks associated with dosage compensation or active transcription that are highly enriched on the male X chromosome are also slightly enriched on the female X chromosome. These findings suggest that the distinct female X chromatin structure is an evolutionary consequence of dosage compensation in males.

## Methods

### Chromatin Preparation

Flies were grown at 25°C on standard cornmeal media. Wild type flies (*y*^1^*w*^67*c*^) for chromatin immunoprecipitation were aged for 5-7 days post-eclosion, sexed and flash frozen. About 0.5 g of sex-sorted adult flies was cross-linked with 1.8% formaldehyde in cross-linking solution (50 mM pH 8.0 HEPES buffer, 1 mM EDTA, 0.5 mM EGTA, and 100 mM NaCl) for 20 minutes at room temperature on a shaker. We confirmed that this procedure cross-linked the DNA by extracting DNA from treated and untreated flies without reversing the cross-linking and performing gel migration retardation experiments. The cross-linked flies were incubated in PBS supplemented with 125 mM Glycine and 0.01% Triton for 5 minutes and then washed twice with PBS and 0.01% Triton. The flies were homogenized and the cells were collected from the pellet after centrifuge at 7500 rpm for 5 minutes at 4°C. The collected cells from adult flies were disrupted in cell lysis buffer (5 mM pH8.0 PIPES buffer, 85 mM Potassium chloride, 0.5% Nonidet P40 and protease inhibitors) for 10 minutes and then resuspended in nuclei lysis buffer (50 mM pH8.1 Tris HCl, 10 mM EDTA, 1% SDS and protease inhibitors) for 20 minutes at 4°C. The nuclear extract was sheared to 200-1000 bp by sonication for 8 minutes on ice (pulsed 8 times for 30 seconds with 30 second intervals) using a Misonix Sonicator 3000 (Misonix, Inc. Farmingdale, NY). After sonication, cell debris was removed by centrifugation and 500 ul of chromatin solution was used for one immunoprecipitation reaction. 5 ul anti-H4K16ac (Upstate, 07-329), 5 ul anti-H3S10ph (Upstate, 05-817) or 3 ul anti-H4K3me2 (Upstate, 07-030) was incubated with the chromatin for 2 hours and then was bound to protein A agarose beads at 4°C overnight. The beads were washed three times with 0.1% SDS, 1% Trition, 2 mM EDTA, 20 mM ph8.0 Tris, 150 mM NaCl; three times with 0.1% SDS, 1% Trition, 2 mM EDTA, 20 mM ph8.0 Tris, 500 mM NaCl; and twice with 10 mM pH8.1 Tris, 1 mM EDTA, 0.25 M LiCl, 1% NP40, 1% sodium deoxycholate. The immunoprecipitated DNA was eluted from the beads in 0.1 M NaHCO3 and 1% SDS. Formaldehyde cross-links were reversed by incubation at 65°C overnight. DNA was purified by phenol-chloroform extraction and ethanol precipitation.

### DNA amplification and Array hybridization

DNA amplification was performed using a Ligation-mediated PCR (LM-PCR) protocol [45] from FlyChip http://www.flychip.org.uk/protocols/chip/lm_pcr.php. 600 ng of amplified DNA (ChIP enriched DNA or input DNA) were then labeled using the Cy3- or Cy5-labeled random nonamers (Trilink Biosciences, San Diego, USA) with Klenow enzymes. The labeled DNA was purified and resuspended in hybridization buffer as described [46]. For hybridizations we used FlyGEM arrays [46], that are spotted arrays with PCR amplicons biased to the 3' ends of annotated genes, which is a suitable platform given that H4K16ac is found to be present along the whole length of target genes with a 3' bias [47] and given our results on shearing patterns at gene models. Labeling with Cy3 and Cy5 random primers was as described [4,46]. There is no dye effect using this end-labeling method [46]. Preliminary ChIP-chip experiments on S2 cells showed no dye effects (not shown). All labeling, hybridization and scanning was done under low ozone conditions. We used Cy5 for ChIP and Cy3 for input in all experiments. Four biological replicates (ChIP vs. input) were performed for H4K16ac in males and three biological replicates were performed for H4K16ac in females. Two biological replicates were performed for H3S10ph and H3K4me2 experiments. Arrays were scanned on an Axon GenePix 4000B (Molecular Devices Corporation, Sunnyvale, CA) and signal for each array elements were extracted with GenePix v.5.1 image acquisition software (Molecular Devices Corporation).

### DNA-Seq and data handling

300 ng of DNA derived from sheared chromatin of female and male adult flies was prepared as outlined for ChIP input controls above. This was used for solexa library preparation using the genomic DNA sample preparation kit (Illumina, San Diego, CA). We size selected libraries (~200 bp) by excision of the appropriate region following agarose gel electrophoresis. We determined library concentration on a Nanodrop spectrophotometer (NanoDrop products, Wilmington, DE) and hybridized 4 pM of adaptor-ligated DNA to the flow cell. DNA clusters were generated using the Illumina cluster station, followed by 36 cycles of sequencing on the Illumina Genome Analyzer. Image analysis and base calling were performed using a manufacturer-provided computational pipeline (version 0.3) including the Firecrest and Bustard applications and sequence reads were then aligned with the *Drosophila melanogaster *assembly (BDGP Release 5, dm3 [48]) using Bowtie (v 0.10.0) [49]. We used only uniquely mapped reads with no more than two mismatches.

We calculated average read coverage depth at each base per Kb per million sequenced base pairs to normalize between samples with slightly different total sequence depth. The average coverage in non-overlapping 1 kb windows were calculated on each chromosome arm for the distribution of sequence coverage in the genome. We also calculated the average coverage for each annotated [50] exon, intron and +/- 3000 bp around the transcription start sites and transcription termination sites in the genome. To test whether the sequenced reads coverage over non-overlapping 1 kb windows or different gene features on individual chromosome arms were significantly different from the whole genome, we performed two sample Kolmogorov-Smirnov tests (KS tests) between random sampled coverage values from non-overlapping 1 kb windows or gene features on individual chromosome arms and the same number of random sampled coverage values from the whole genome. The coverage for gene features on male X chromosome was multiplied by 2 to correct for the different number of X chromosome in male. The sample size for random sampling was 1000 for all chromosome arms except for the 4^th ^chromosome (sample size = 200) and the permutation procedure was repeated 100 times. The distribution of *P *values from the 100 permutation KS tests were used to determine whether the genes features on one particular chromosome arms were significant different from the gene features in the genome. For visualization purpose, we calculated the average coverage along different gene features in 100 overlapping windows with widow size as 1/10 of the whole region for individual genes and then plotted the moving averaged coverage along different gene features for genes on the X chromosome and different autosome arms separately.

### Microarray data handling

All microarray data were processed and analyzed in R/Bioconductor [51] package limma. For the ChIP-chip studies, the log_2 _ratio between two channels for individual arrays were median-centered and then all the arrays were normalized using quantile normalization based on the input channel across arrays while assuring that the input channel has the same empirical distribution among arrays. Background corrections were then applied to exclude array elements with intensities less than the average intensities of control elements (designed against non-*Drosophila *DNA) in both channels for downstream analysis. The average intensities of the control elements were also subtracted from the intensities of every array element on two channels separately. Duplicated array elements were merged by calculating the average ratio from duplicated probes. The consistency between biological replicates was checked by the density scatter plot of the input and ChIP DNA intensities among all replicates (see Additional file 1, 2 for H4K16ac ChIP replicates in male adult files, Additional file 3, 4 for H4K16ac ChIP replicates in female adult files and Additional file 5 for other histone marks). The raw and normalized data were inspected to make sure that normalization was appropriate (see Additional file 6).

The comparisons of the distribution of ChIP/input ratios or ChIP input intensities among different chromosome arms were performed using two sample Kolmogorov-Smirnov tests (KS tests) and the *P *values were adjusted using the Bonferroni correction for multiple-comparison. The maximum adjusted *P *value was truncated at 1.0. KS tests and the Bonferroni corrections were applied to ChIP/input ratios from individual replicates and averaged ratios to test the significance of X chromosome in different replicates. Unless otherwise noted, all ChIP/input ratios used in the figures were averaged across all the biological replicates. Spearman's Rank Correlation was used to test the relationship between H4K16ac level and all other histone modifications in males and females. To test the correlation among different histone marks, we also used the ChIP/input ratio ranks in each ChIP sample for K-means clustering analysis with 2 nodes using the Euclidean similarity metric. X chromosome genes and autosome genes were clustered separately and the order of ChIP samples was fixed as indicated in the figure in both clustering analysis. Data were clustered and visualized using Cluster3.0/Tree-View. The expression data for sex-sorted whole adults and for gonadectomized male and female carcasses were from GEO accession number GSE6640 and GSE442. The expression values used for the correlation between marks and expression were averaged expression values from all replicates in each dataset. For the correlation between expression and different histone marks, genes with ChIP/input ratio percentile rank >90% were used as genes with enriched marks and genes with ChIP/input ratio percentile rank <10% were used as genes with depleted marks to assure similar sample size for different histone marks. The list of genes with male-, female- or non-biased expression in adult flies were got from a previous study [52] and the list of genes with sex-biased expression in gonadectomized carcasses were produced from the data set GSE442 using the same analysis approach [52].

### Data sources

All DNA-Seq and ChIP-chip data and full platform description are available at GEO under accession number GSE15593.

## Authors' contributions

YZ designed the project, collected and analyzed the data and wrote the manuscript. BO designed the experiments, provided guidance in interpreting the results and wrote the manuscript. Both authors read and approved the final manuscript.

## Supplementary Material

Additional file 1 **Density scatter plots of H4K16ac ChIP input DNA intensities in male adult flies**. H4K16ac ChIP input DNA intensities (log_2_) between all biological replicates in male adult flies, plotted against each other (high data density in blue). The corresponding *R*^2 ^values are shown in each graph. Click here for file

Additional file 2 **Density scatter plots of H4K16ac ChIP DNA intensities in male adult flies**. H4K16ac ChIP enriched DNA intensities (log_2_) between all biological replicates in male adult flies, plotted against each other (high data density in blue). The corresponding *R*^2 ^values are shown in each graph. Click here for file

Additional file 3 **Density scatter plots of H4K16ac ChIP input DNA intensities in female adult flies**. H4K16ac ChIP input DNA intensities (log_2_) between all biological replicates in female adult flies, plotted against each other (high data density in blue). The corresponding *R*^2 ^values are shown in each graph. Click here for file

Additional file 4 **Density scatter plots of H4K16ac ChIP DNA intensities in female adult flies**. H4K16ac ChIP enriched DNA intensities (log_2_) between all biological replicates in female adult flies, plotted against each other (high data density in blue). The corresponding *R*^2 ^values are shown in each graph. Click here for file

Additional file 5 **Density scatter plots of H3K4me2 and H3S10ph ChIP-chip intensities in male and female adult flies**. H3K4me2 and H3S10ph ChIP input (A) or enriched DNA (B) intensities (log_2_) between biological replicates, plotted against each other (high data density in blue). The corresponding *R*^2 ^values are shown in each graph. Click here for file

Additional file 6 **Density MA plots of normalized ChIP experiments**. ChIP/input ratio (log_2_) versus average ChIP and input intensity (log_2_) plots for H4K16ac ChIP (A-B), H3S10ph ChIP (C-D) and H3K4me2 ChIP (E-F) in male and female adult flies (high data density in blue). Each MA plot represents data from one biological replicate of each ChIP data set. Click here for file
