# Supplementary figures and images for: An evolutionary consequence of dosage compensation on Drosophila melanogaster female X-chromatin structure?
Source: BMC Genomics. 2010 Jan 5;11:6. doi: 10.1186/1471-2164-11-6 (PMC2820458; doi:10.1186/1471-2164-11-6)

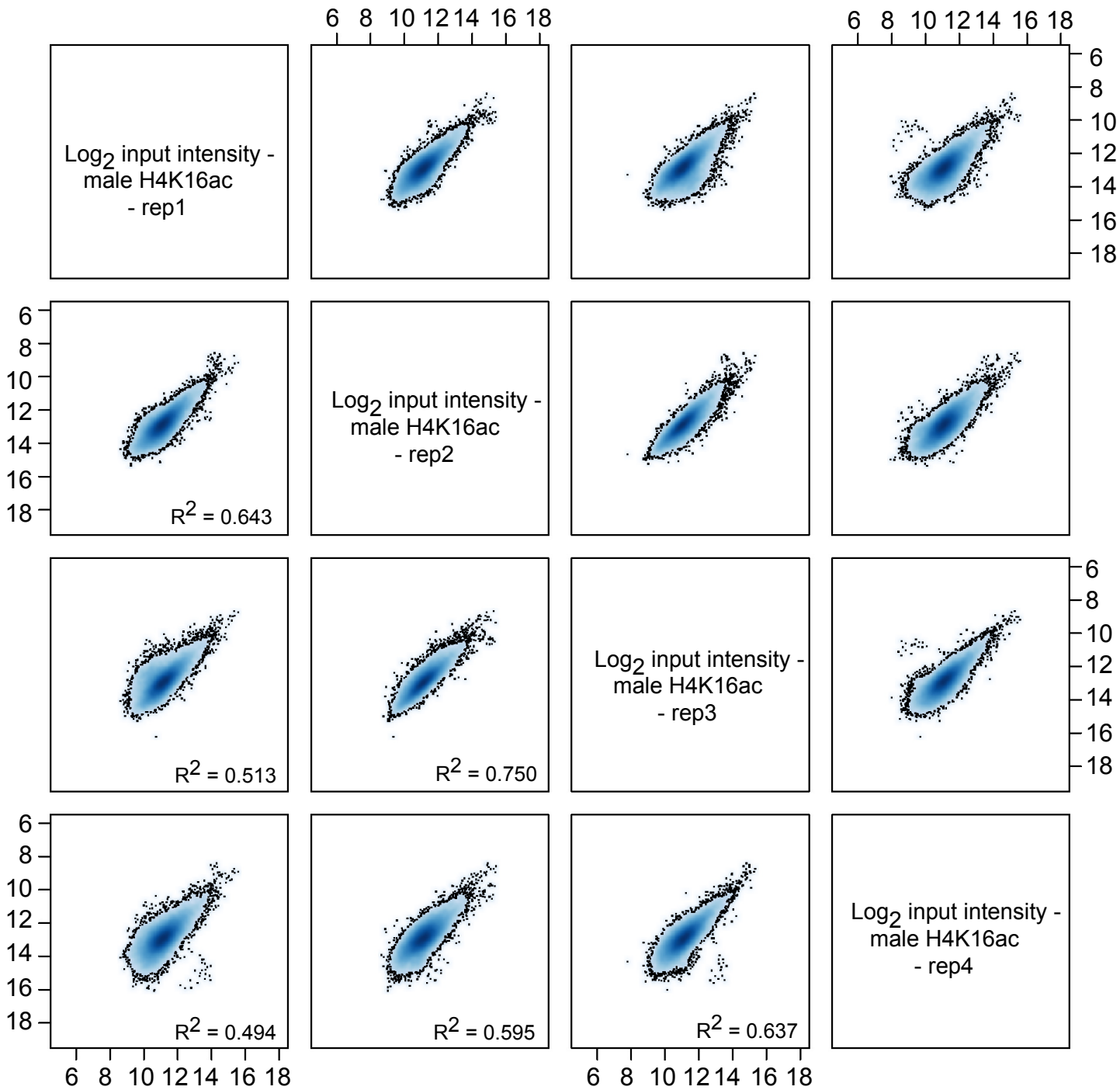

Supplement: Additional file 1 — Density scatter plots of H4K16ac ChIP input DNA intensities in male adult flies. H4K16ac ChIP input DNA intensities (log2) between all biological replicates in male adult flies, plotted against each other (high data density in blue). The corresponding R2 values are shown in each graph. [file 1471-2164-11-6-S1.PDF]

**A**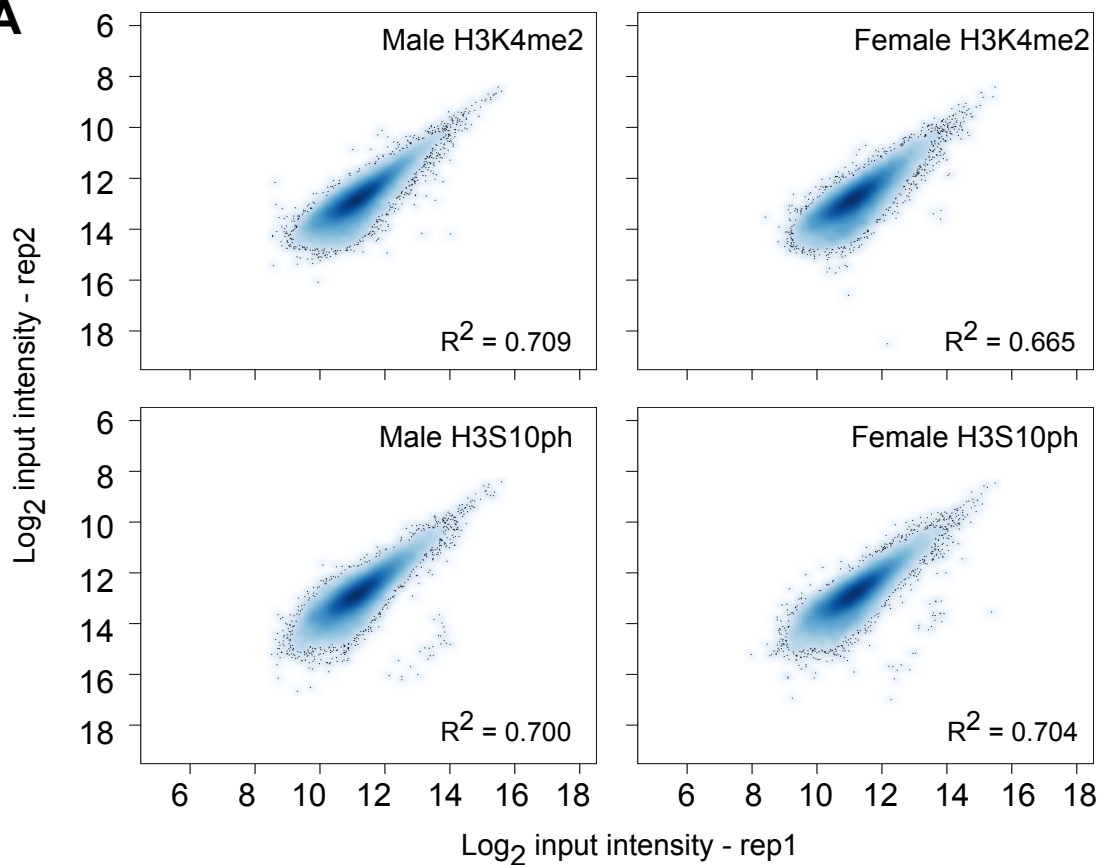**B**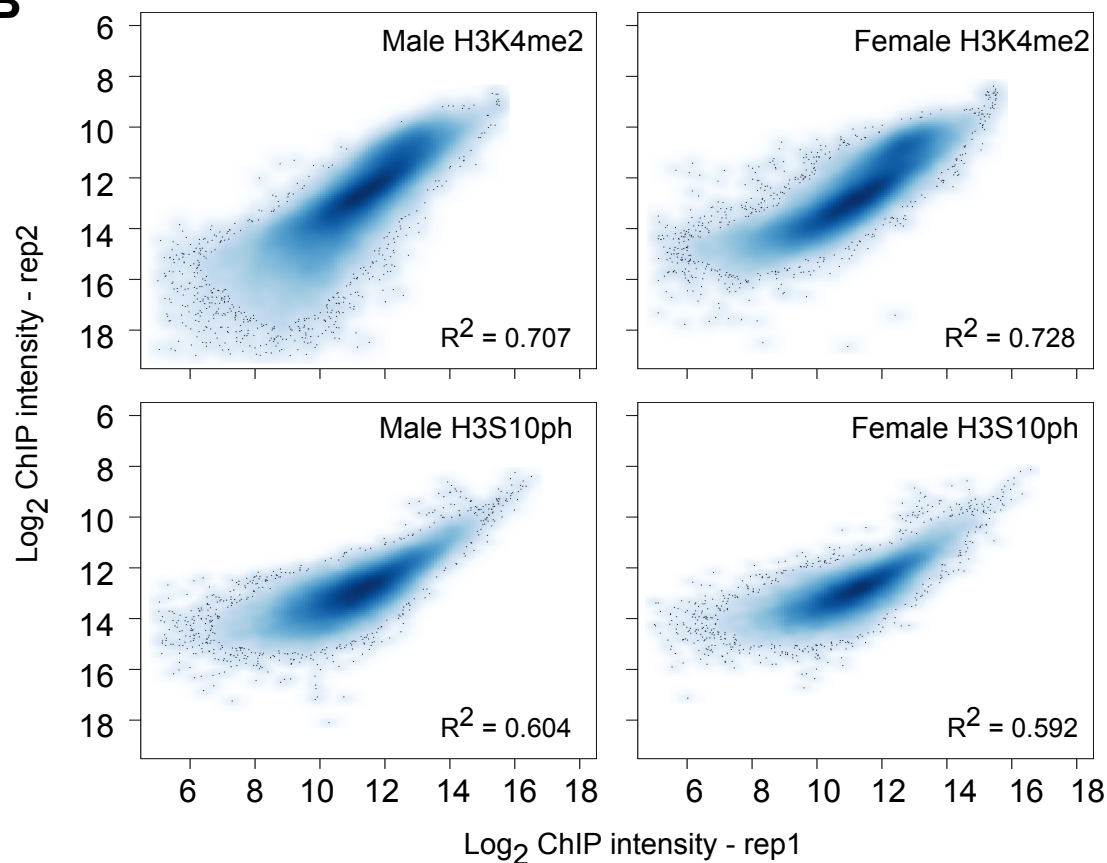

Supplement: Additional file 5 — Density scatter plots of H3K4me2 and H3S10ph ChIP-chip intensities in male and female adult flies. H3K4me2 and H3S10ph ChIP input (A) or enriched DNA (B) intensities (log2) between biological replicates, plotted against each other (high data density in blue). The corresponding R2 values are shown in each graph. [file 1471-2164-11-6-S5.PDF]

Male

Female

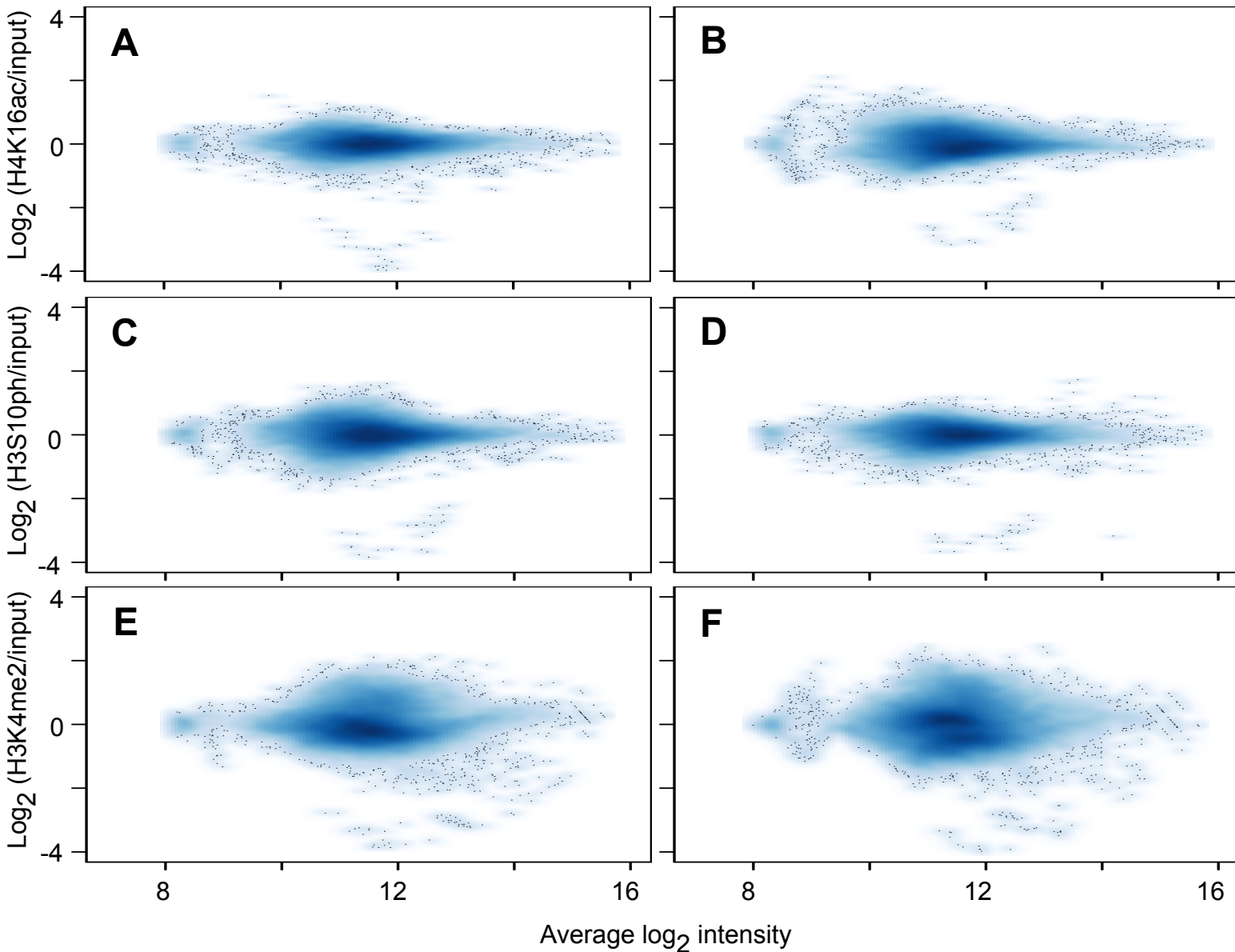

Supplement: Additional file 6 — Density MA plots of normalized ChIP experiments. ChIP/input ratio (log2) versus average ChIP and input intensity (log2) plots for H4K16ac ChIP (A-B), H3S10ph ChIP (C-D) and H3K4me2 ChIP (E-F) in male and female adult flies (high data density in blue). Each MA plot represents data from one biological replicate of each ChIP data set. [file 1471-2164-11-6-S6.PDF]
