# Supplementary material for: An evolutionary consequence of dosage compensation on Drosophila melanogaster female X-chromatin structure?
Source: BMC Genomics. 2010 Jan 5;11:6. doi: 10.1186/1471-2164-11-6 (PMC2820458; doi:10.1186/1471-2164-11-6)
Supplement: Additional file 2 — Density scatter plots of H4K16ac ChIP DNA intensities in male adult flies. H4K16ac ChIP enriched DNA intensities (log2) between all biological replicates in male adult flies, plotted against each other (high data density in blue). The corresponding R2 values are shown in each graph. [file 1471-2164-11-6-S2.PDF]

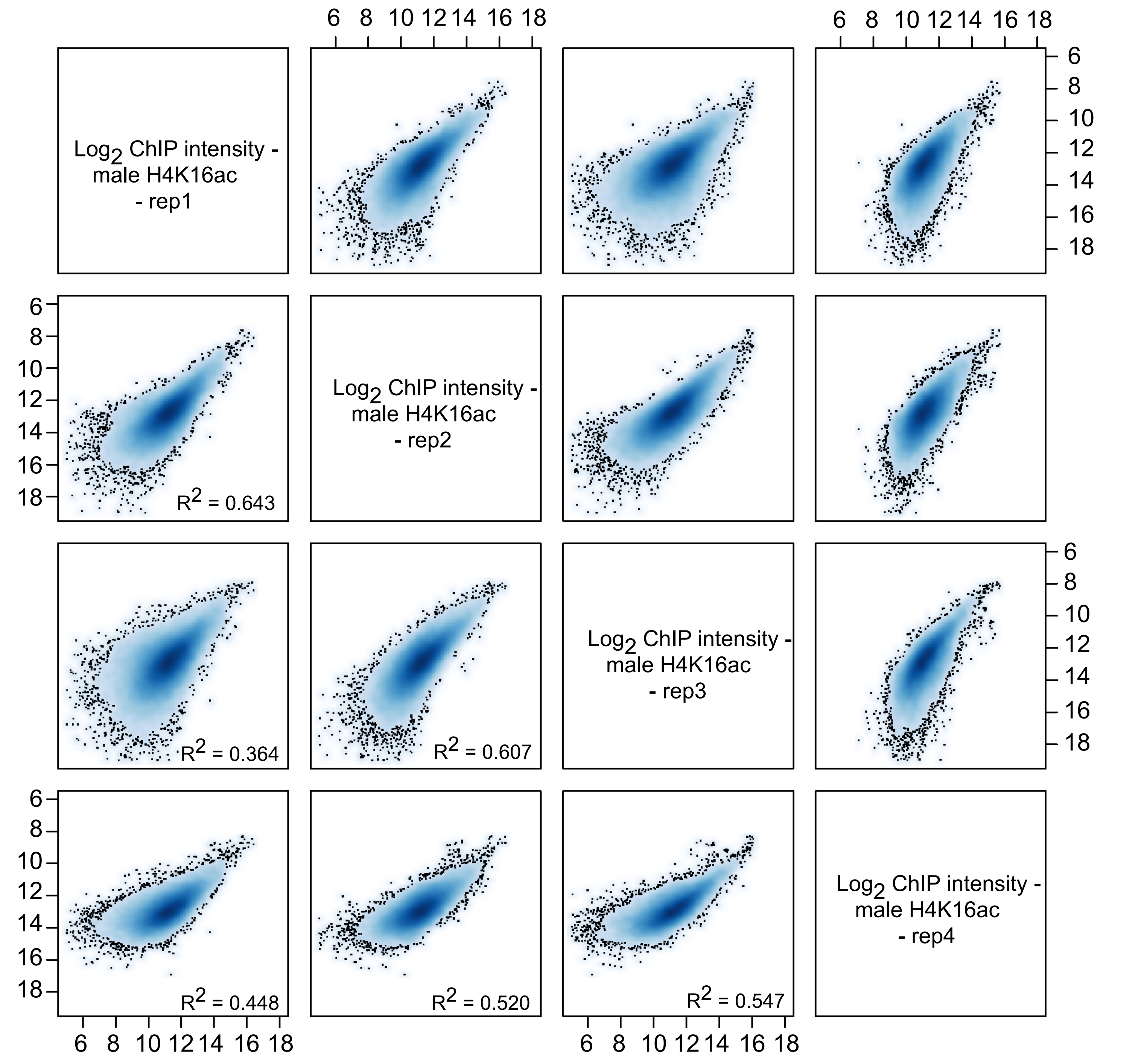

Log2 ChIP intensity - male H4K16ac - rep1
Log2 ChIP intensity - male H4K16ac - rep2
Log2 ChIP intensity - male H4K16ac - rep3
Log2 ChIP intensity - male H4K16ac - rep4
R2 = 0.643
R2 = 0.364
R2 = 0.607
R2 = 0.448
R2 = 0.520
R2 = 0.547
6 8 10 12 14 16 18
